# Factors associated with cervical cancer screening participation among migrant women in Europe: a scoping review

**DOI:** 10.1186/s12939-020-01275-4

**Published:** 2020-09-11

**Authors:** Patrícia Marques, Mariana Nunes, Maria da Luz Antunes, Bruno Heleno, Sónia Dias

**Affiliations:** 1 NOVA National School of Public Health, Public Health Research Centre, Universidade NOVA de Lisboa, Lisbon, Portugal; 2 Comprehensive Health Research Center (CHRC), Universidade NOVA de Lisboa, Lisbon, Portugal; 3 ESTeSL (Instituto Politécnico de Lisboa), Lisbon, Portugal; 4 APPsyCI – Applied Psychology Research Center Capabilities & Inclusion, ISPA, Lisbon, Portugal; 5 NOVA Medical School, Universidade NOVA de Lisboa, Lisbon, Portugal

**Keywords:** Migrants and transients, Emigrants and immigrants, Uterine cervical neoplasms, Early detection of Cancer

## Abstract

**Background:**

Cervical cancer screening has been effective in reducing incidence and mortality of cervical cancer, leading European countries to implement screening programs. However, migrant women show lower screening participation compared to nationals. This scoping review aims to provide a synthesis of the growing evidence on factors associated with participation in cervical cancer screening among migrant women in Europe.

**Methods:**

Electronic peer-reviewed databases were searched in November 2019 for studies on factors related to the participation of migrants in cervical cancer screening conducted in EU/EFTA countries, using comprehensive search expressions. Retrieved articles were screened and those eligible were selected for data extraction. Quantitative and qualitative studies were included. Factors were classified in barriers and facilitators and were divided into further categories.

**Results:**

Twenty out of 96 articles were selected and analyzed. Factors associated with participation in cervical cancer screening were classified in categories related to sociodemographic, healthcare-system, psychological, migration, knowledge, language, and cultural factors. Lack of information, lack of female healthcare providers, poor language skills, and emotional responses to the test (especially fear, embarrassment and discomfort) were the most reported barriers to cervical cancer screening. Encouragement from healthcare providers and information available in migrants’ languages were frequently stated as facilitators. Results on the role of sociodemographic factors, such as age, education, employment and marital status, are the most conflicting, highlighting the complexity of the issue and the possibility of interactions between factors, resulting in different effects on cervical cancer screening participation among migrant women. Several identified barriers to screening are like those to access to healthcare services in general.

**Conclusions:**

Efforts to increase migrant women’s participation in CCS must target barriers to access to healthcare services in general but also specific barriers, including cultural differences about sexuality and gender, past traumatic personal experiences, and the gender and competences of healthcare professionals performing CCS. Healthcare services should strengthen resources to meet migrants’ needs, including having CCS information translated and culturally adapted, as well as healthcare providers with skills to deal with cultural background. These findings can contribute to improve CCS programs among migrant women, reducing health disparities and enhancing their overall health and well-being.

## Introduction

Cervical cancer ranks as the second most common cancer in females in Europe [1]. In 2018, the incidence of cervical cancer reached 15.9 and mortality was 4.6 per 100,000 women aged 15–64 in the region [2]. In nearly all the cases, cervical cancer is characterized by a persistent infection caused by one or multiple different genotypes of Human Papillomavirus (HPV) [3]. Early detection of the cancerous lesions through preventive strategies and tools leads to a positive prognosis and a higher chance of the patient being fully cured [4].

Cervical cancer screening (CCS) is a public health intervention that includes identifying and inviting an eligible population at risk of cervical cancer, providing a screening test to detect HPV-virus or abnormal cervical cells, providing diagnostic tests to women who screen-positive and direct these women for treatment. The screening test requires that a healthcare professional performs a gynecological examination and collects a cervical sample [5]. According to the International Agency for Research on Cancer (IARC), CCS programs have been shown to be an effective strategy to reduce the incidence of the disease [4, 6–8]. Evidence shows that cervical cancer mortality was reduced in European countries that implemented organized CCS programs [9]. Reduction in cervical cancer incidence and mortality in opportunistic screening ranged from 10 to 60% worldwide [8].

By 2016, 22 out of 28 EU member states had started planning or implementing publicly-funded, organized CCS programs [10]. However, participation in CCS programs has been suboptimal, with low coverage of the population [4]. According to the report of the Council Recommendation on the implementation of cancer screening in 19 European countries, 59.2% of the annual target women aged 30–59 years were invited for screening and mean participation rate was 50.7% (ranging from 11.6 to 67.7%) [1]. This means that there is still a significant part of the eligible female population who is not screened regularly for cervical cancer.

Migrant women are a vulnerable group to cervical cancer. Several studies conducted in high-income countries found higher incidence rates of cervical cancer among migrant women compared to native counterparts [11–15]. Additionally, lower participation rates in CCS have been found among migrant women, which increases the risk of being diagnosed in later stages of the disease, with negative impact on treatment outcomes [5, 16].

Indeed, underuse of health services among migrant populations has been well documented in the literature, including for early detection measures [17–19] such as CCS [20]. Lower access and utilization of health services, especially for preventive and primary care, have been associated to barriers such as economic constraints, undocumented migration status, poor living conditions and social integration, and limited knowledge about the host country’s healthcare system and migrants health rights [19, 21–24]. In addition to these barriers, other factors seem to influence participation in CCS among migrants, and are related to limited knowledge on cervical cancer and screening [25–27], emotional attitudes toward CCS, such as fear and embarrassment [25], cultural and religious factors [26], and lack of culturally-adapted responses of the health services for provision of health care to migrant women, such as interpreters and female healthcare providers [26, 27].

Official data estimate that in mid-2019 the number of international migrants in Europe was around 82.3 million, of whom 51.4% were women [28] mostly in reproductive age [29]. Despite the evidence that many migrant women are particularly vulnerable to cervical cancer and are under-screened, knowledge on the factors influencing their participation in CCS is limited and sparse. An overview of the body of evidence currently available on the barriers and facilitators to CCS is crucial to inform strategies to increase migrant women participation in CCS and reduce the prevalence of the disease in this population. The aim of this study is to provide a synthesis of the growing evidence on factors associated with participation in CCS among migrant women in Europe, that can be useful for recommendations to increase participation. A scoping review was performed as it is an appropriate approach to explore the available evidence, to provide trends in the literature and to identify key factors related to a concept [30–32]. The core research question guiding this review was “What are the factors influencing participation in cervical cancer screening among migrant women in Europe?”

## Methods

This study followed the PRISMA extension for scoping reviews (PRISMA-ScR) framework [33].

### Data sources and search strategy

The search was conducted in the following electronic peer-reviewed databases: CINAHL, EMBASE, PsycINFO, PubMed, Scopus, and Web of Science. “Migrant” was defined as *“any person who is moving or has moved across an international border or within a State away from his/her country of origin”,* regardless of the reason or duration of the movement [34], including documented migrants, undocumented migrants, asylum seekers, and refugees among others. “Europe” was defined as the 28 member states of the European Union (EU) and the four states of European Free Trade Association (EFTA), as they share similar legislation regarding cancer screening and implementation of population-based CCS practices [35].

Search expressions combined controlled vocabulary and free text and included relevant keywords and Medical Subject Heading (MeSH) terms related to the main topics, namely “migrant”, “cervical cancer”, “screening”, and “Europe”. Boolean operator “AND” was used to combine the main topics and “OR” was used to combine keywords of each of the topics. Search expressions were adjusted to each database’s specifications **(see** Additional file 1**).** The search strategy was developed by the first author with the collaboration of a health information specialist.

The search involved three steps:

1. A pilot search was conducted in three databases (PubMed, Web of Science and Scopus) to refine the search strategy and ensure it was precise enough to include relevant literature;
2. A complete analysis using the final search strategy was conducted in all databases on 11th November 2019. All the articles retrieved were stored in Mendeley reference management software, and duplicates were removed.
3. Supplementary electronic searches were conducted by manual searching the references of included papers.

### Study selection

The present review included studies that focus on participation in CCS among migrant women, from the perspective of migrant women and/or healthcare providers and/or stakeholders that work with migrants living in EU/EFTA countries. Original research articles, regardless of the publication year or the study type, published in English, Portuguese, or Spanish were included to have a broader view of the existing evidence. Articles were excluded if they did not present data about migrant women, were not conducted in the EU/EFTA countries, or if the full version of the article was not accessible, after attempting to contact the authors. Grey literature was not included due to resources and time limitations.

Two authors performed an independent assessment of 20% of the included articles, whereas the remaining articles were screened by a single author. In case of disagreements, a third reviewer was contacted to reach an agreement.

### Data extraction and synthesis of results

Data was extracted by one of the authors, and included: author, year of publication, country where the study was conducted, study design, study population, sample size, and factors associated with CCS participation **(see** Additional file 2**).**

Factors associated with CCS participation were categorized into barriers (i.e. factors reducing CCS participation) and facilitators (i.e. factors enhancing CCS participation) and were further divided in categories. The construction of the initial categories was developed based on literature review [36] and was completed with additional categories that emerged through data analysis. Each category aggregated subcategories according to the data analyzed. Categories were organized from the most frequently cited to the least frequently cited among the studies retrieved and presented in a table form.

## Results

### Sources of evidence

**Fig. 1:**
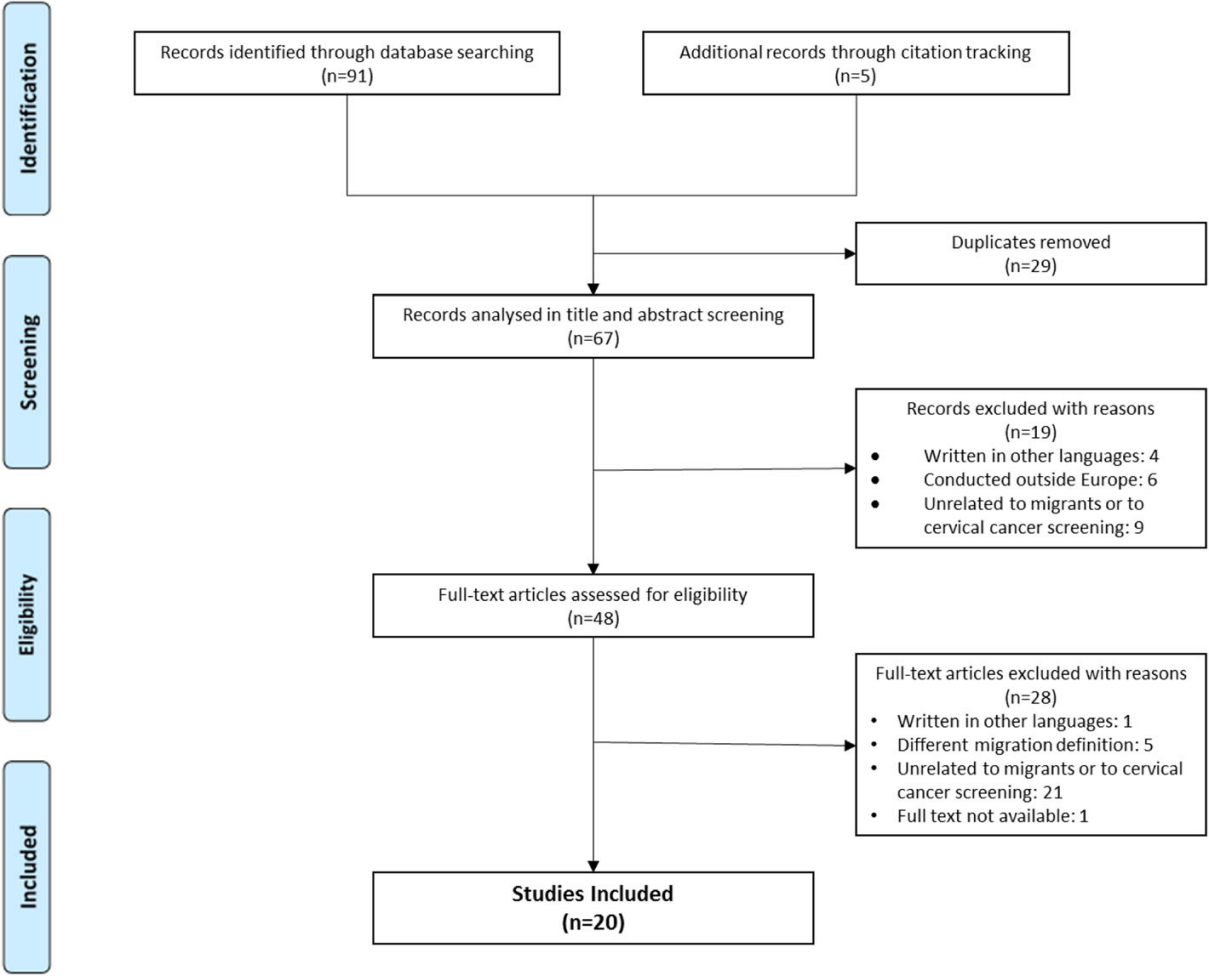
PRISMA flowchart of the search process and data selection. CCS: cervical cancer screening

Figure 1 presents the PRISMA flowchart of the search and selection process of the studies. Through electronic databases, 91 peer-reviewed articles were found - 44 in PubMed, 32 in EMBASE 11 in Scopus, three in Web of Science, and one in PsycINFO. No results were found in CINAHL. Five additional records were retrieved by citation tracking of the included papers, among which four were not indexed to any database at the time the search was conducted. After removing duplicates (*n* = 29), 67 articles were screened for title and abstracts. A total of 19 articles were excluded for not meeting the inclusion criteria. Of the 48 articles eligible for full-text screen, 28 were excluded, mostly for not being related to migrants or cervical cancer screening. The final review included 20 original research articles.

### Characteristics of selected studies

Table 1 shows the main characteristics of the studies included in the review. Studies were published between 2009 and 2019 and were written in English except for one article in Spanish. Most of the studies were conducted in northern Europe (Norway, Sweden, Finland, and Denmark), and in southern Europe (Spain and Italy). A total of ten studies were quantitative, the majority being cross-sectional (*n* = 7), and three being longitudinal. Among the ten qualitative studies, five used focus groups, one used interviews, and four used both methods.

**Table 1:** Study characteristics

Study	Year	Country	Study Type	Study population	Sample Size
Abdullahi et al. [37]	2009	England	Qualitative Focus Groups and Interviews	Migrant women (Somali)	Migrants: 50
Otero et al. [38]	2011	Spain	Qualitative Interviews	Healthcare providers (Midwives)	Healthcare providers: 10
Azerkan et al. [12]	2012	Sweden	Quantitative Longitudinal	Migrant women (Not specified) Native women	Total: 2,621,802 Migrants: 445,547
Jackowska et al. [39]	2012	England	Qualitative Focus Groups and Interviews	Migrant women (Polish, Romanian, and Slovak) Healthcare providers (Nurses, consultants, GP practice manager, gynecologist, and healthcare assistant) Stakeholders (Advocate working with migrants)	Migrant women: 42 Healthcare providers: 10 Stakeholders: 1
Rodríguez-Salés et al. [40]	2013	Spain	Quantitative Cross-sectional	Migrant women (Not specified) Native women	Total: 1,562,968 Migrants: 251,679
Olsson et al. [41]	2014	Sweden	Qualitative Focus Groups	Stakeholders (Doulas)	Stakeholders: 13
Azerkan et al. [42]	2015	Sweden	Qualitative Focus Groups	Migrant women (Danish and Norwegian)	Migrants: 40
Grandahl et al. [43]	2015	Sweden	Qualitative Focus Groups	Migrant women (Middle Eastern, African, Asian, and East European)	Migrants: 50
Akhagba [44]	2017	Poland	Qualitative Focus Groups	Migrant women (African)	Migrants: 12
Bianco et al. [45]	2017	Italy	Quantitative Cross-sectional	Migrant women (European, African, Asian, and American)	Migrants: 464
Comparetto et al. [46]	2017	Italy	Quantitative Cross-sectional	Migrant women (Not specified) Native Women	Total: 69,459 Migrants: 7339
Gallo et al. [47]	2017	Italy	Quantitative Longitudinal	Migrant Women (High income countries and low- and middle-income countries) Native women	Total: 1,610,855 Migrants: 227,061
Gele et al. [27]	2017	Norway	Qualitative Focus Groups	Migrant women (Pakistani and Somali)	Migrants: 35
Idehen et al. [48]	2017	Finland	Quantitative Cross-sectional	Migrant women (Russian, Somali, and Kurdish)	Migrants: 620
Møen et al. [49]	2017	Norway	Quantitative Cross-sectional	Migrant women (Western Europe, Eastern Europe, Asia, Africa, and South America) Native women	Total: 1,321,632 Migrants: 152,800
Addawe et al. [50]	2018	Norway	Qualitative Focus Groups and Interviews	Migrant women (Somali)	Migrants: 57
Idehen et al. [51]	2018	Finland	Quantitative Cross-sectional	Migrant women (Russian, Somali, and Kurdish) Native women	Total: 973 Migrants: 537
Møen et al. [52]	2018	Norway	Qualitative Focus Groups and Interviews	Healthcare providers (General practitioners, midwives, and gynecologists)	Healthcare providers: 33
Barrera-Castillo et al. [53]	2019	Spain	Quantitative Cross-sectional	Migrant women (Not specified) Native women	Total: 8944 Migrants: 886
Hertzum-Larsen et al. [54]	2019	Denmark	Quantitative Longitudinal	Migrant women (Western, and Nonwestern) Native women	Total: 610,907 Migrants: 57,329

Study populations were diverse across the studies. In total, eight studies included migrant and native women, eight studies included only migrant women, two included healthcare providers, one included stakeholders, and one included migrants, healthcare providers and stakeholders. Several studies focus on specific groups of women (mostly Somali, Russian, Kurdish, and Pakistani women), and seven studies include migrant women irrespective of place of birth. Healthcare providers included in the studies were general practitioners, gynecologists and midwives. Among stakeholders, one study included doulas and another study included consultants and advocates.

### Factors associated with cervical cancer screening participation

Seven main categories were used to classify both barriers and facilitators of CCS participation, providing a wider and complete framework to characterize the factors associated with participation in CCS. The categories related to sociodemographic, healthcare-system, psychological, migration, knowledge, language, and cultural factors.

### Barriers to CCS participation

All 20 studies mentioned barriers to the participation in CCS among migrant women.

Table 2 provides an overview of the barriers documented in the articles, classified in the different categories.

**Table 2:** Barriers to cervical cancer screening participation among migrant women

Factors	Study	Participants’ country of origin
*Sociodemographic*		
Older age groups	[47] [46] [49] [40]	Low- and middle-income countries Not specified Eastern Europe Not Specified
Younger age groups	[47] [49] [48]	High income countries Asia Russia
Being unmarried	[47] [51] [54] [53]	Low- and middle-income countries Somalia Not specified Not specified
Being married/cohabiting	[49] [51] [42]	Asia and South America Russia and Kurdistan Denmark and Norway
Not having children	[48] [54] [42]	Kurdistan Not specified Denmark and Norway
Having children	[42]	Denmark and Norway
Low social support	[42] [53] [44] [41]	Denmark and Norway Not specified Africa Not Specified
Low educational level	[47] [48]	Not specified Kurdistan
Being unemployed	[51] [48] [54]	Russia and Kurdistan Kurdistan Not specified
Being employed	[51]	Somalia
No insurance	[53] [38]	Not specified Not specified
Low socioeconomic status	[27] [49] [54] [38] [41]	Pakistan and Somalia Not specified Not specified Not specified Not Specified
Very high socioeconomic status	[47]	Not specified
Having a smaller household	[51]	Kurdistan
Having a bigger household	[51]	Russia and Somalia
Living in urban areas	[49] [51]	Not specified Russia and Kurdistan
Living in rural areas	[51] [48]	Somalia Somalia
*Healthcare system*		
Not having a female healthcare provider	[27] [49] [50] [37] [43] [52]	Pakistan and Somalia Not specified Somalia Somalia Not specified Not specified
Not having a gynecologist doing the screening test	[52] [39]	Not specified Poland, Slovakia, Romania
Perception of the screening as impersonal	[42]	Denmark and Norway
Long waiting time in healthcare services	[42] [44] [39]	Denmark and Norway Africa Poland, Slovakia, Romania
Lack of time and/or information from healthcare providers	[27] [52]	Not specified
Poor hygiene in healthcare services	[39]	Poland, Slovakia, Romania
Lack of access to healthcare services	[46]	Not specified
Negative relationship with healthcare provider	[27]	Pakistan and Somalia
Unprofessional healthcare providers	[42] [50] [39]	Denmark and Norway Somalia Poland, Slovakia, Romania
Healthcare providers lack of skills to work with migrant women	[52]	Not specified
Having a migrant healthcare provider	[49]	Western and Eastern Europe, Asia
Unaccustomed to preventive healthcare	[43] [41]	Not specified Not Specified
Lack of regular medical checkups	[48] [54] [41]	Russia, Somalia, Kurdistan Not specified Not Specified
*Psychological*		
Fear of the screening test	[44] [37] [43] [52] [39] [41]	Africa Somalia Not specified Not specified Poland, Slovakia, Romania Not Specified
Fear of the test result/fear of cancer	[42] [50] [37] [39] [41]	Denmark. Norway Somalia Somalia Poland, Slovakia, Romania Not Specified
Emotional discomfort about the screening test	[42] [44] [50] [43] [39] [41]	Denmark, Norway Africa Somalia Not specified Poland, Slovakia, Romania Not Specified
Fatalistic views about cancer	[50] [41]	Somalia Not Specified
Lack of trust in healthcare services	[27] [50] [39] [41]	Pakistan, Somalia Somalia Poland, Slovakia, Romania Not Specified
Negative past experiences in healthcare services	[27] [44] [37] [41]	Pakistan and Somalia Africa Somalia Not Specified
Experiencing sexual assault	[42]	Denmark, Norway
Experiencing female genital mutilation	[27] [50]	Somalia Somalia
Other life priorities	[42] [37] [52] [41]	Denmark, Norway Somalia Not specified Not Specified
Lack of time	[50] [37] [39] [41]	Somalia Somalia Poland, Slovakia, Romania Not Specified
*Migration-related*		
Country of birth	[12] [47] [46] [40] [45]	Not specified Africa, Asia Not specified South-Central Asia, Western Europe, South-Eastern Asia, North America Not specified
Short length of stay in the country	[49] [48] [54] [42] [39] [45]	Not specified Russia Not specified Denmark, Norway Poland, Slovakia, Romania Not specified
Long length of stay in the country	[49]	Western Europe, Asia, Africa, South America
Older age at migration	[12]	Not specified
Re-immigration	[54]	Not specified
Attending cervical cancer screening in home country	[44] [39]	Africa Poland, Slovakia, Romania
*Knowledge-related*		
Lack of information about cervical cancer screening	[27] [44] [38] [50] [37] [43] [39] [41]	Pakistan, Somalia Africa Not specified Somalia Somalia Not specified Poland, Slovakia, Romania Not Specified
Lack of information about cervical cancer	[27] [50] [43]	Pakistan, Somalia Somalia Not specified
Low perceived need of screening	[42] [50] [37]	Denmark, Norway Somalia Somalia
Lack of information regarding the healthcare services	[42] [38] [43] [39]	Denmark, Norway Not specified Not specified Poland, Slovakia, Romania
*Language-related*		
Language difficulties	[27] [48] [44] [37] [52] [39] [41]	Pakistan, Somalia Russia Africa Somalia Not specified Poland, Slovakia, Romania Not Specified
Family member/male interpreter during the screening test	[27] [52]	Pakistan, Somalia Not specified
Not having an interpreter during the screening test	[37] [41]	Somalia Not Specified
*Cultural*		
Cultural differences	[42] [39]	Denmark, Norway Poland, Slovakia, Romania
Social stigma about women’s health	[43] [41]	Not specified Not Specified
Religious beliefs	[27] [50]	Pakistan and Somalia Somalia

#### Sociodemographic-related barriers

Age appears as one of the most common sociodemographic determinants of low participation in CCS. However, studies offer contradictory evidence on the role of age on screening participation. Older age (over 40 years of age) was associated with lower participation in CCS in four studies [40, 46, 47, 49]. It is suggested that higher attendance to CCS among younger migrants (below 40 years of age) might be related to their desire to get pregnant, which leads to increased contact with healthcare services [46, 47]. Yet, in other research, younger age was associated with lower participation in CCS among Russian [51] and Asian [49] migrants. One of the studies showed that younger women from high income countries (HIC) participate less in CCS, in opposition to the trend found in low and middle income women, where older women are the ones that participate the least [47]. This might be related to the migratory pressure, where women from low and middle income countries migrate at a younger age and, therefore, might be screened opportunistically for cervical cancer as they look for healthcare support for fertility reasons [46, 47].

Parity and pregnancy were also considered factors influencing CCS participation, but also with conflicting results [42, 48, 54]. Quantitative data suggests that having no children is associated with lower attendance of screening among migrants from either Western and Nonwestern countries [48, 54]. A qualitative study offers a nuanced perspective on the influence of pregnancy in the use of screening. Younger women stated that the desire to get pregnant encouraged them to take the screening test as cervical cancer was a dangerous disease that could compromise pregnancy. Conversely, since pregnancy is a period marked by frequent contact with healthcare professionals, some women felt that they could monitor their health without doing the screening test. Additionally, after giving birth priorities change and CCS is not a priority [42].

Several studies show that screening participation may be influenced by the marital status of the women, but its effect is not consistent across studies. Some studies suggest that unmarried migrants are less likely to participate in CCS [47, 51, 53, 54], while others show that married women participate the least [42, 49, 51]. These conflicting results might be related to different cultural norms in different countries of origin, or generational effects. For example, in one of the studies, older women mentioned that participation in CCS tend to be lower when their husbands do not think CCS is important, while younger women stated their partners were more likely to be supportive of CCS participation [42]. Indeed, having social support, either by the partner or husband, or even by family or friends has been showed to have positive impact on CCS participation [42]. Lack of social support was linked to postponing or avoiding screening [44, 53].

Lower education has been negatively associated with CCS participation [47, 48], as well as being unemployed [48, 51, 54] and having a low income [49, 54]. There are exceptions. Being employed was associated with lower participation among Somali women [51]. Some migrant women with a very high socioeconomic position participated less in organized screening. This may reflect a higher utilization of private medical care [47]. Having no private insurance or no insurance at all also seems to be a barrier to CCS among migrants living in Spain, as shown in two Spanish studies [38, 53].

Household setting and household size also have been found to be associated with CCS adherence. Living in urban areas was associated with lower participation in CCS among European, Asian and African migrants [49, 51], which is thought to be the result of a better integration of migrant women in rural communities and a higher proximity with healthcare providers of those regions as they have less patients [49]. However, among Somali women living in rural areas, the opposite trend is observed [48, 51]. These two studies indicate that these women face difficulties in accessing healthcare services due to long distances and lack of transportation.

#### Healthcare system barriers

One of the barriers to participation in CCS most reported among migrant women [27, 37, 43, 49, 50, 52] and healthcare providers [52] is the lack of availability of female healthcare providers to perform the screening test when the patient requires it. In two qualitative studies, Somali women stated that having a male doctor performing the exam is not acceptable within their cultural and religious values, and believed that it might compromise their modesty and virginity [37, 50]. Feelings of shame, awkwardness and shyness have been reported when a male healthcare provider is present [27]. Healthcare providers are also aware of this barrier and male doctors mention that they often send migrant women to a female colleague to do the screening test [52]. Among women from Eastern Europe a barrier to screening participation is having a practitioner who is not a gynecologist perform the test. This might reflect differences in screening practices between the country of origin and the host country; in Eastern European countries usually a gynecologist performs the test [39, 52].

Lack of access to healthcare services, mainly by not being registered in the country’s healthcare system, reduces the likelihood of participating in CCS [46]. Migrant woment that were invited to an organized CCS in Norway by an invitation letter perceived it as impersonal [42]. Long waiting times, for both the exams and the results, also leads women to postpone cervical screening [39, 42, 44]. Eastern European women (Polish, Slovak, and Romanian) in a English study referred poor hygiene in healthcare services as a barrier to participation in screening in England [39].

Negative attitudes from healthcare providers prevent women from attending cervical screening, either by unprofessional treatment [39, 42, 50] or failing to establish a good patient-healthcare provider relationship [27]. Some women felt discriminated against because of their migrant status or language skills [39], or felt that their beliefs and culture were disrespected [50]. This can be exacerbated by a lack of skills to work with migrant women, as referred to by healthcare providers [52]. A study showed that having a GP with a migrant background was associated with lower CCS participation among European and Asian women [49]. Also, some women felt like their healthcare providers do not give them enough time to discuss their issues, or that the appointment time was insufficient to address their issues [27, 52].

Women originating from countries with different health systems and preventive practices might not be accustomed to preventive healthcare services [41, 43] or attending medical checkups regularly [41] and, therefore, they become unaware of their needs regarding CCS.

#### Psychological barriers

CCS attendance may be affected by women’s specific individual characteristics related to their experiences, emotions and behaviors. Fear of the screening procedure was one of the main barriers leading women to postpone screening, stated by migrants [37, 39, 43, 44], healthcare providers [52], and doulas [41]. Female migrants stated that they fear the test procedure and materials, and fear the pain they associate with the exam [37, 39]. Fear of the result of the test also stopped women from taking the screening test. A large number of women did not want to know if they had cancer, and therefore they decided to not take the test [37, 39, 41, 42, 50]. Other psychological and emotional barriers included shyness, embarrassment, defenselessness, and discomfort for exposing their body [39, 41–44, 50]. Emotional responses can also show up as fatalistic views about cancer, believing that it cannot be prevented nor cured and therefore it would be pointless to do the screening test [41]. Some women also believe that it would be God’s will if they have cancer or not so they did not attend the screening test [50].

Lack of trust in healthcare services also prevents many women from using them [27, 39, 41, 50]. This distrust in the services is most likely the result of negative past experiences and fear of misdiagnosis [27, 37, 39, 41, 44]. Negative past experiences, such as pain, bleeding, or unprofessionalism from healthcare providers has been shown to be a barrier either if the women experienced it herself or if she was told about it [37].

Past traumatic experiences may also act as a barrier to attending CCS. Previous experiences of sexual assault [42], or suffering from female genital mutilation [27, 50] may lead women to postpone attendance as these experiences might cause discomfort, fear or shame.

Lack of time and having other priorities were also referred [37, 39, 41, 42, 50, 52]. Healthcare providers [52] and doulas [41] observe that women do not prioritize CCS, or even if they find it important, they do not have spare time to attend it due to other priorities, included children [37, 41, 50], tasks related to their migration status [42], or work [39].

#### Migration-related barriers

There is contradictory evidence on the association between country of birth and CCS participation among studies. Immigrant women from low and middle income countries showed a lower screening participation when compared with immigrant women from high income countries in an Italian study [47]. Asian women, particularly those coming from China, were the ones with the lowest participation rates [40, 45–47]. Being born in an African country was associated with lower participation in an Italian study [47], but in a Spanish study, Sub-Saharan African women are the ones with the highest participation rate [40]. A cohort study conducted in Sweden showed that migrants from Australia and New Zealand are the ones who participate the least in CCS, and that the low participation might be the result of past negative experiences in their home countries [12].

A shorter length of stay in the hosting country is associated with lower screening participation, and this might be related to migration-related stress, lack of knowledge about healthcare services in the host country, or difficulties with the language [45, 49, 54]. One example of this situation are transient migrants, who after having arrived to the host country often move around within and between countries and, therefore, might not be invited for organized screening nor be screened opportunistically [39, 42]. However, in a Norwegian study, it was found that Western European that stay in the country for longer are less likely to attend to screening. This could be related to their preference of attending screening in their home country [49].

Other migration-related factors with a negative impact on CCS participation include older age at migration [12], not being the first time a woman migrates (re-immigration) [54]. Also, some women showed preference to attend CCS in their home countries [39, 44].

#### Knowledge-related barriers

Low knowledge about CCS was largely referred to as a barrier to CCS participation by migrants [27, 37, 43, 44, 50], healthcare providers [38, 39], and doulas [41]. Women that expressed limited knowledge about cervical cancer often do not attend CCS [27, 43, 50]. Also, women frequently state that they do not need CCS in the absence of symptoms, showing a low risk perception of the disease [27, 37, 42, 50]. In the included studies, women expressed a wide range of knowledge about cervical cancer and cancer screening [37, 39, 44, 50]. Despite the hosting country, evidence shows there is a high level of unawareness regarding CCS among Somali women [37, 44, 50]. Additionally, a study from the England showed that the level of awareness of CCS varied according to the countries of origin [39]. The lack of knowledge of how the healthcare system of the host country works leads to low service utilization, resulting in lower attendance to CCS [38, 39, 42, 43].

#### Language-related barriers

Language difficulties was one of the most commonly cited barriers to CCS in the studies, either by migrants [27, 37, 39, 44, 48], healthcare providers [52], or doulas [41]. Most healthcare providers communicate in the host country’s native language, in which migrants are seldom fluent [27, 37]. In addition, information about healthcare services and specifically about CCS is frequently only provided in the host country’s native language, which prevents migrant women to get properly informed about screening practices in the country [37, 44]. In a cross-sectional study conducted in Finland [51], Russian women participated more frequently in CCS than Somali or Kurdish women and it was proposed that this difference might result from a similarity between Russian and host country’s language which could facilitate communication and knowledge diffusion among that specific population.

The type of interpreters used during appointments can also be a barrier to CCS. Most migrant women end up using their husbands or other relatives as interpreters and this, per se, works as a barrier because their privacy is compromised and women might not feel comfortable to talk about intimate issues in the presence of their relatives [27]. This problem is also perceived by healthcare providers [52]. On the other hand, frequently when women are provided with interpreters to help them with the appointments, many are not satisfied with the quality of the interpreters which may prevent them to further attend screening appointments as shown in a study with Somali women in Camden, England [37].

#### Cultural barriers

Different cultural backgrounds between migrants and host society can also act as a barrier to CCS [39, 42]. In some cultural contexts, social stigma about women’s health exists [41, 43]. Especially among Muslim women, for whom talking about reproductive health might be a taboo topic, leaving women feeling uncomfortable with approaching these issues [27, 41, 43]. In certain countries women’s health is undervalued, and that prevents women from attending preventive healthcare services and being screened for cervical cancer [43]. Also, there is the belief in some communities that only married women should have gynecological examinations. An unmarried woman might be stigmatized for going to the gynecologist, related to social prejudice on the need of an unmarried woman having the exam [43].

Religious beliefs and values also have a strong role in the decision of participating in CCS. In a study with Pakistani and Somali women, some participants stated that religion protects them against diseases and therefore they do not need to be screened [27]. The same study states that this belief might be more related to the fear of the disease rather than a religious imposition. Another study with Somali women stated that the invasiveness of this test could interfere with beliefs of modesty that are intrinsically related to their religion and they therefore seem to be reluctant to take the screening test [50].

### Facilitators of CCS participation

Among the 20 articles retrieved, 11 explored facilitators to participation in CCS [27, 37–39, 42, 43, 46–48, 50, 52]: eight were qualitative studies, one was conducted with healthcare providers, and one included migrants, healthcare providers, and stakeholders. Facilitators were classified under three categories: healthcare system, knowledge and language-related, and cultural factors, which are presented from the most cited in the articles to the least cited. Table 3 summarizes the facilitators found in the literature.

**Table 3:** Facilitators to cervical cancer screening participation among migrant women

Facilitating factors	Study	Participants’ country of origin
*Healthcare System*		
Healthcare providers’ explanations and encouragement	[27] [50] [37] [52] [39]	Pakistan, Somalia Somalia Somalia Not specified Poland, Slovakia, Romania
Having had a recent medical appointment	[46] [48]	Not specified Russia, Somalia, Kurdistan
Good organization and promotion of screening programs	[38] [43] [39]	Not specified Not specified Poland, Slovakia, Romania
Regular invitations to cervical cancer screening	[27] [42] [38] [39]	Pakistan. Somalia Denmark, Norway Not specified Poland, Slovakia, Romania
*Knowledge and language-related*		
Providing information about cervical cancer and screening	[27] [50] [37] [39]	Pakistan, Somalia Somalia Somalia Poland, Slovakia, Romania
Providing information leaflets/invitation letters in women’s mother languages	[27] [47] [50] [43] [39]	Pakistan, Somalia Not specified Somalia Not specified Poland, Slovakia, Romania
Providing experienced interpreters	[27] [37] [52]	Pakistan, Somalia Somalia Not specified
Collaboration with stakeholders to promote CCS	[37] [41]	Somalia Not Specified
*Cultural*		
Dialogue about cultural issues	[50] [52]	Somalia Not specified

#### Healthcare system facilitators

Well organized programs, that are easy to navigate, well promoted and with no costs associated were mentioned as facilitators both in qualitative and quantitative studies [38, 39, 43]. Regular invitations and reminders seemed to be appreciated and were also mentioned as ways to increase CCS participation [27, 38, 39, 42]. Women who were invited regularly also manifested a feeling of security and that their health is being checked [42].

Healthcare providers encouragement, by being proactive in inviting women to do CCS [37, 50], investing enough time on their patients to explain the procedure [27, 37, 52], and being careful and respectful while doing the test [39] were healthcare provider behaviors that stood out as facilitators in CCS among studies. Also, having a recent general practitioner’s or gynecological appointment is associated with a higher attendance to CCS, as these practitioners might incentivize those women to attend CCS during the appointments [46, 48].

#### Knowledge and language-related facilitators

Providing migrant women with relevant, and easy to understand information about cervical cancer and screening has been mentioned as a key facilitator to increase CCS participation [27, 37, 39, 50]. Some studies suggest that this information should be provided by medical doctors [39] or through workshops and community activities [50], Also, information should be accessible in places frequented by the target women [50].

Additionally, it was shown that activities in collaboration with community stakeholders could also help to increase participation. One study described an intervention of doulas in a specific community of migrants and the results were highly positive [41]: doulas worked as a link between healthcare professionals and migrant women and provided them information and help regarding CCS, in migrants’ native language, which facilitated the process of participation. Another study with Somali women said they preferred education about CCS provided by Somali speakers [37].

Offering information leaflets and sending invitation letters in migrants’ native language was also one of the most highlighted facilitators [27, 39, 43, 47, 50]. Another facilitator is having bilingual healthcare providers or having interpreters experienced in medical contexts to assist the appointments and help with the communication between the healthcare providers and women [27, 37, 52]. Additionally, the use of simpler language, body language, translated materials, and anatomic models to provide explanations about CCS by healthcare providers is a strategy that can be used to increase participation [52].

#### Cultural facilitators

One study mentioned that women were more open to participate in CCS if they knew that healthcare providers would consider their preferences regarding, for instance, having the CCS test taken by a female doctor [50]. Another facilitator is the perception that healthcare workers try to understand and to overcome cultural barriers that they might face when working with migrant women [52].

## Discussion

Participation in CCS among migrant women is influenced by several factors. This is a complex issue, in which a wide range of factors may affect independently, synergistically, or antagonistically the participation in CCS among migrant women.

Results of this scoping review show that language barriers [27, 37, 39, 41, 44, 48, 52], lack of information about CCS [27, 37–39, 41, 43, 44], unavailability of female healthcare providers to do CCS [27, 37, 43, 49, 50, 52], and emotional factors [37, 39, 41–44, 50, 52] were the most commonly reported barriers to CCS, whereas healthcare providers encouragement of regular CCS [27, 37, 39, 50, 52], regular reminders and CCS invitations [27, 38, 39, 42], and having information in migrant women’s native languages [27, 39, 43, 47, 50] were the facilitators most reported.

Many of the documented barriers to CCS are similar to those faced by migrants in healthcare services in general. For migrants arriving at a new country, accessing healthcare services is challenging - differences in the healthcare structure of the host country, language, low health literacy and cultural differences – highlighting what are described as barriers to health services utilization among migrants [18, 19]. The role of health services-related factors in CCS participation shown in this scoping review highlights that migrant-friendly healthcare services can positively impact migrants’ healthcare access and participation in CCS. Our findings show that strategies aiming to provide adequate medical provision adapted to migrants’ specific needs and helping them navigate the healthcare services could be the key to improve their access to healthcare services and CCS access, in line with the World Health Organization and the International Organization for Migration recommendations [16, 55].

There are, however, specific factors that are particularly relevant when it comes to promoting participation in a CCS program. CCS can be a quite invasive test that requires sampling from the uterine cervix. In addition, sexual health can be a sensitive issue for many women. As found in this study, women’s health is considered a taboo issue for some women, and many of them are not comfortable talking about it even with their healthcare providers [41, 43]. The gender of the health professional was found to be one of the main barriers to CCS, as women might feel uncomfortable with a male healthcare provider and might avoid CCS practices when no female provider is available. This barrier is consistent across studies [56–58], highlighting the importance of gender. Also, our results suggest that some migrant women, especially coming from European countries, may expect having a gynecologist doing CCS, rejecting to do the test if it is performed by other healthcare professional [39, 52]. To engage migrants in CCS programmes it seems important to offer the option of having a female healthcare provider or a gynaecologist for issues related to sexual health.

Most women, despite their country of origin, are more likely to participate in CCS when recommended by a trusted healthcare provider [56, 59]. Limited knowledge among migrants about CCS seems to be a challenge, as reported by both migrant women and healthcare providers. Evidence shows that the greater the knowledge about CCS, the highest the level of participation [60]. However, knowledge is influenced by other factors, such as cultural and religious factors, misconceptions about the risk of developing the disease. Emotional responses to the screening test such as fear or embarrassment may also discourage women participating in CCS or even seeking information about CCS [56, 61, 62]. Language difficulties also limit the access to information, compromising participation in CCS as well [63]. Also, in some situations, women need their husbands or family members to be interpreters in medical appointments [27, 52] which may make them feel uncomfortable to open up with their medical doctor and avoid doing the CCS. An additional obstacle is related to past traumatic experiences of sexual assault or genital mutilation [27, 42, 50] which may be difficult to address as it requires additional competencies and, for instance, psychological support for those women.

The development and implementation of a cultural-sensitive healthcare system requires allocating appropriate resources and healthcare personnel prepared to provide the care needed by these underscreened groups [17, 19, 58]. Based on this study’s findings, some key recommendations can be highlighted. It is important that the information about CCS is delivered in a linguistically adapted and culturally sensitive way to target these groups. Skilled healthcare workers to intervene with different cultural backgrounds to better meet the migrants needs and encouraging medical doctors to promote CCS in appointments with migrant women may also play a role in increasing CCS participation. However, interventions aiming only to increase knowledge may be insufficient [64]. A multi-factor-oriented strategy can be a more effective approach to increase participation in CCS besides targeting individual-level factors or barriers. Establishing partnerships with community workers can be an approach to reduce the gap between healthcare providers and migrant populations. These professionals can help migrants navigating through health system and can also inform healthcare providers of specific needs of these populations. Some examples of successful community-based interventions regarding CCS documented elsewhere include: partnerships with doulas sharing language and cultural background with migrant women [41] or faith-based community organizations [65], and interventions based on community education activities to increase awareness of CCS and help women navigate through the system [66].

One of the major limitations of this review is that studies about some European countries with high percentage of migrants (e.g. Luxembourg, Switzerland, Greece) [67] were not found. Grey literature was not included in the study so some relevant information could be overlooked. Language restrictions were applied in this review; therefore, potentially relevant articles might have been excluded. Studies selection and data extraction were mainly performed by one author due to resources constraints; nevertheless, the authors are confident that the quality of the data was not compromised as 20% of the included abstracts was independently assessed by two authors and concordance was 100%.

The major strength of this scoping review relies in the comprehensive search conducted in different databases using a robust search expression, and complemented by citation tracking of the documents, based on an existing conceptual framework [36]. The studies included represent the context among different EU/EFTA nations and different migration populations which provides a wide perspective about the topic. Additionally, most CCS programs in these countries share similar guidelines [35] which means that factors identified may also be relevant to other EU/EFTA countries.

## Conclusions

Migrant women continue to show lower CCS participation rates when compared to native women. The findings of this scoping review reinforce that strategies targeting the improvement of access to healthcare services in general can have a positive effect also on CCS participation. Additionally, specific barriers related to access to CCS must also be addressed and include social stigma, gender and cultural-based values, religious beliefs, fear and embarrassment, and past traumatic personal experiences. Developing a migrant-friendly healthcare system is crucial to increase migrants’ participation in preventive care. Healthcare services should strengthen resources to meet migrants’ needs, including having CCS information translated and culturally adapted, as well as healthcare providers with competences to deal with cultural background and different experiences. Having female professionals available to do the screening test may already be put in place with existing resources. These findings can be used by policymakers, healthcare providers, and community workers to improve CCS programs, increasing migrant women’ participation, reducing health disparities and enhancing their overall health and well-being.

## Supplementary information

**Additional file 1.** Search strategy for each database (conducted in 11th November, 2019).

**Additional file 2.** Data extraction form.

## Data Availability

All data generated or analyzed during this study are included in this published article.
