# Supplementary material for: Factors associated with cervical cancer screening participation among migrant women in Europe: a scoping review
Source: Int J Equity Health. 2020 Sep 11;19:160. doi: 10.1186/s12939-020-01275-4 (PMC7488650; doi:10.1186/s12939-020-01275-4)
Supplement: Supplementary file 1 — Additional file 1. Search strategy for each database (conducted in 11th November, 2019). [file 12939_2020_1275_MOESM1_ESM.docx]

**Additional File 1** - Search strategy for each database (conducted in 11^th^ November, 2019)

|  | **Search expression** | **Nº Studies** |
| --- | --- | --- |
| **PubMed** | (uterine cervical neoplasms[MeSH Terms]) OR cervical  cancer[Title/Abstract]  AND  ((papanicolaou test[MeSH Terms]) OR cytology[MeSH Terms]) OR  early detection of cancer[MeSH Terms]  AND  ((((((transients and migrants[MeSH Terms])) OR (emigrants and  immigrants[MeSH Terms])) OR migrant[Title/Abstract]) OR  emigrant[Title/Abstract]) OR immigrant[Title/Abstract]) OR  refugee[Title/Abstract]  AND  Europe[MeSH Terms] | 44 |
| **Web of Science** | TOPIC: ("uterine cervical neoplasms") OR TOPIC:  ("cervical cancer")  AND  TOPIC: ("papanicolaou test") OR TOPIC: (cytology) OR  TÓPICO: ("early detection of cancer")  AND  TS=(migrants) OR TS=(emigrants) OR TS=(immigrants) OR  TS=(refugees)  AND  TOPIC: (Europe) | 3 |
| **EMBASE** | exp uterine cervix tumor/ OR cervical cancer.mp.  AND  exp Papanicolaou test/ OR exp cytology/ OR exp early cancer diagnosis/  AND  "emigrant*".m_titl. OR "migrant*".m_titl. OR "immigrant*".m_titl. OR "refugee*".m_titl.  AND  exp Europe/ | 32 |
| **CINAHL** | SU cervix neoplasms OR SU cervical cancer  AND  SU papanicolaou test OR SU cytology OR SU early detection of cancer  AND  SU ( transients and migrants ) OR SU ( emigrants and immigrants ) OR TI migrants OR TI emigrants OR TI immigrants OR TI refugee  AND  SU Europe | 0 |
| **PsycINFO** | uterine cervical neoplasms.mp. OR cervical cancer.mp.  AND  papanicolaou test.mp. OR exp Cytology/ OR exp Cancer Screening/  AND  emigrant.mp. OR exp Human Migration/ OR exp Immigration/ OR undocumented immigrant.mp. OR exp Refugees/  AND  Europe.mp. | 1 |
| **Scopus** | ("uterine cervical neoplasms").kw OR ("cervical cancer").ti.abs.kw  AND  ("papanicolaou test").kw OR (cytology).kw OR ("early detection of cancer").kw  AND  ("Transients and Migrants").kw OR ("emigrants and immigrants").kw OR (migrant).ti.abs.kw OR (emigrant).ti.abs.kw OR (immigrant).ti.abs.kw OR  (refugee).ti.abs.kw  AND  (Europe).kw | 11 |
