# Supplementary material for: Factors associated with cervical cancer screening participation among migrant women in Europe: a scoping review
Source: Int J Equity Health. 2020 Sep 11;19:160. doi: 10.1186/s12939-020-01275-4 (PMC7488650; doi:10.1186/s12939-020-01275-4)
Supplement: Supplementary file 2 — Additional file 2. Data extraction form. [file 12939_2020_1275_MOESM2_ESM.docx]

**Additional File 2 –** Data extraction form.

| **Study** | **Year** | **Country** | **Study Type** | **Study population** | **Sample Size** | **Population characteristics** | **Factors associated with CCS** |
| --- | --- | --- | --- | --- | --- | --- | --- |
| Reference of the article | Year of publication of the study | Country where the study was conducted | Type of study and main data collection strategies used  (E.g.: Qualitative study - Focus Groups) | Description of the population studied  (E.g.: Migrants, healthcare professionals, stakeholders) | Number of participants of each group defined in study population | Description of the following characteristics of the study population, if available:  Age, country of origin, occupation | Factors associated with CCS participation among migrant women organized in barriers and facilitators |
